# The relationship between intimate partner violence and maternal practices to correct child behavior: a study on women in Egypt

**DOI:** 10.5249/jivr.v2i1.17

**Published:** 2010-01

**Authors:** Koustuv Dalal, Stephen Lawoko, Bjarne Jansson

**Affiliations:** ^*a*^Linköpings Universitet, Department of Medical and Health Sciences, Centre for Medical Technology Assessment & Division of Social Medicine and Public Health Sciences, SE-581 83 Linköping, Sweden.; ^*b*^Karolinska Institutet, Department of Public Health Sciences, Division of Social Medicine , SE 171 76, Stockholm, Sweden.

## Abstract

**Background:**

This paper scrutinizes the association between maternal practices to correct child behavior and the mothers' exposure to and attitudes towards intimate partner violence (IPV).

**Methods:**

Nationally representative data comprising 14 016 married women were retrieved from the Egyptian Demographic and Health Survey, 2005. Data on practices used to correct child behavior, exposure to IPV, attitudes towards IPV were our primary interest. Data were analyzed using Chi-square test and logistic regression.

**Results:**

The majority of the mothers reported use of violent methods, like shouting (90.6%), striking (69.1%) and slapping (39.3%) to correct child behavior. Seven percent of the mothers used only the explanation option. Exposure to physical IPV and tolerant attitudes towards IPV were associated with an augmented risk of using violent methods (shouting, striking or slapping) to correct child behavior. On the other hand non-tolerant attitudes towards IPV were associated with increased likelihood of sole use of the explanation method.

**Conclusions:**

We thus recommend the implementation of local parental education programs focusing on communicative skills to reduce IPV and related child abuse.

## Introduction

Child abuse is now acknowledged as a global public health problem.^1^ Abused children are likely to face socioeconomic and health problems in their transition to adulthood.^2-4^ According to the World Health Organization, 57 000 children under the age of 15 years died in the year 2000 as a result of homicide.^5^ In general, between 80-98 percent of children in their homes are victims of physical punishment and at least 30 percent are victims of severe punishment in which implements are used.^6^ In Egypt 37 percent^7^ of all children face severe physical punishment from their parents, comparable with observations from the Republic of Korea, 45 percent^8^, Romania, 50 percent^9^, Ethiopia, 64 percent^10^, India, 36 percent^5^, and the Philippines, 21 percent.^5^Besides physical abuse, children are also often victims of emotional and psychological abuse. Data from Egypt indicate that such abuse occurs among 72 percent of Egyptian children, which is comparable with observations from Chile, 84 percent, India, 70 percent, Philippines, 82 percent and the U.S.A, 85 percent.^5^

Child abuse has a significant impact on children’s wellbeing, including several psychological and somatic symptoms,^11-13^  medical and psychiatric problems like depression^14-16^ eating disorders,^17^ anxiety,^16-17^  posttraumatic stress disorder (PTSD)^16,18,19^ chronic pain syndromes^18,20-22^ fibromyalgia^13,23,24^chronic fatigue syndrome^23^ and irritable bowel syndrome.^21,25,26^ Moreover, the effects of such abuse are likely to be long-term. As adults, formerly abused children report poor health status^26-28^  make more use of health care facilities^29^ and opt for risk-taking behaviors including smoking^30,12^ alcohol and drug abuse^12,17,27^ and unsafe sex.^12,27^

A number of studies have been undertaken to explore the risks and elicit factors regarding child abuse. In this regard, poverty, lower socioeconomic status, lower level of parental education, large families, low family income and young parental age have been suggested as plausible risks or eliciting factors.^31-35^ Moreover, studies suggest further that a history of spouse abuse in the family may be related with an augmented risk of child abuse^31-32,36-41^and that those fathers practicing IPV are more prone to abuse their children than the mothers.^42^

In recent years there has been a growing concern about the association between child abuse and spousal violence.^43,44^ Data indicate that children are at higher risk of physical abuse and/or punishment when their mothers are also abused in the home.^34,35,39,40^ However, there are limited data on the different facets of abuse that women are exposed to and how these are related to child abuse. Most studies have focused on the association between physical spousal violence and child abuse.^31,43,44^ The relationship between emotional/ sexual abuse of women and abuse of their children has not been studied to the same degree. Moreover, particular emphasis has been put on studies scrutinizing whether the perpetrator of spousal violence (in most cases men) is also prone to abuse their children.^31,43,45^ Whether the victims of spousal violence (in most cases women) are also likely to abuse their children has only received minimal attention in the research. Moreover, no study has focused on methods used (violent and non-violent) to correct child behavior among mothers who are also often victims of IPV. Another gap in the research pertains to a lack of studies from developing countries in Africa. Most studies in the field are from the industrialized countries. Considering that societies may differ socio-culturally with regards to how children are brought up, further studies are warranted to investigate these issues in dissimilar societal contexts. Finally, the factors associated with a non-violent approach to correcting child behavior have, to the best of our knowledge, not been investigated previously. Not all parents use violence to correct child behavior. A pertinent hypothesis in the field is that a history of violence in the family (e.g. spousal violence) or a lack of it could in part account for differences between parents in the use of violent or non-violent methods to correct child behavior. Yet, no study has been undertaken, as far as we know, regarding the association between spousal abuse and a non-violent approach to child discipline. This paper will address some of these limitations.

More specifically, the objective of this paper is to study the association between exposure to IPV (i.e. physical, sexual and emotional spousal abuse), attitudes towards IPV and violent/non-violent approaches to correcting child behavior among women in Egypt. Possible confounding variables (e.g. age, education and urban/rural residency) will also be considered in the analysis.

## Methods

**a. The Egyptian Interim Demographic and Health Survey:**

The Egyptian Demographic and Health Survey (EDHS) of 2005 is the fifth full-scale survey in the DHS series, which are performed on a five-yearly basis. It was conducted by the Ministry of Health and Population and National Population Council of Egypt and sponsored by the U.S. Agency for International Development (USAID).

**b.Sample design:**

 A systematic sample of 22 807 households was selected for interview from 682 primary sampling units (PSUs) in the EDHS 2005. PSUs were selected from 289 towns and 393 villages in six major subdivisions (Urban Governorates, urban Lower Egypt, rural Lower Egypt, urban Upper Egypt, rural Upper Egypt and the Frontier Governorates) in Egypt. A total of 19 565 women, between 15 and 49 years were interviewed from the selected households giving a response rate of over 99 percent. For this study however, only women with children (n = 14016) were included.

**c. Questionnaire:**

A comprehensive questionnaire covering demographic and health issues was administered. The questionnaire included the demographic and socioeconomic backgrounds of the women and their husbands, empowerment and social status, reproductive history, attitudes towards family planning, maternal health care, antenatal and delivery care, child care and nutrition, child mortality, immunization and health, awareness of and precautions against sexually transmitted diseases, female genital mutilation, attitudes towards wife abuse and exposure to violence among women and children. For the current study, the questions concerning practices used by mothers to correct child behavior, spousal violence and the mothers’ attitude towards wife beating were of primary interest. Possible confounders, i.e. demographic variables were included.

**d. Measures:**

**1-Dependent variable**

Practices used by mothers to correct child behavior constituted the dependent variables in this study. Mothers were asked to indicate which of the following methods they used to correct child behavior: explaining to children, shouting at them, striking or slapping their children. In each case the response alternatives were ‘yes’ or ‘no’ and mothers could choose one or several of these methods. In addition, a variable reflecting the use of any physical violence (i.e. either striking or slapping or both) was created based on the responses.

**2-Independent variables**

Data on intimate partner violence (IPV) were assessed using the conflict tactics scale.^45^Respondents were asked whether, over the previous year, they had experienced any sexual, physical and emotional violence. Sexual violence was assessed by asking respondents whether they had experienced forced sexual intercourse by husbands. Physical violence was assessed by asking respondents whether they had been pushed, slapped, punched, kicked, attacked with a weapon by husbands or whether any attempt to strangle or burn them had been made. Finally emotional violence was assessed by asking respondents whether they had been humiliated or threatened verbally or with some kind of implement or weapon by husbands.

Data on respondents’ attitude towards wife beating were collected by asking respondents whether they felt physical violence from husbands was justified if the wife: went out without permission, neglected children, argued with their husband, refused sex with their husband and burnt the food. In each case respondents had three alternatives: ‘yes’, ‘no’ and ‘do not know’. For the current analyses, responses in the latter two categories constituted a single category.

The demographic variables included were age, residential area, education, literacy level, and current occupational status.

**e.Ethical issues:**

The DHS procedure (e.g. with regard to organization and sampling methods) and instruments used have received ethical approval from the Institutional Review Board of Opinion Research Corporation (ORC), Macro International Incorporated. In addition, the recommendations of WHO, concerning the assurance of women’s safety while at the same time maximizing disclosure ^46^ were followed through the provision of sufficient training and support to the field workers enrolled. The respondents had the opportunity to give informed consent, and were given a guarantee of privacy.

**f. Statistical analyses:**

Differences in values of the dependent variable (explaining behavior and violence against children) between participants in different categories of the explanatory variables (i.e. IPV, attitude to wife beating and demographics) were assessed by chi-square. To control for possible confounding, the independent associations between the dependent and independent variables were assessed using logistic regression. The magnitude and direction of associations were expressed in odds ratios (ORs). For all the statistical analyses, a significance level of p< .05 was employed. For the statistical analysis SPSS version 15 was used.

## Results

**a. Proportion of mothers using specific practices to correct child behavior by demographic variables:**

Of the 14 016 participants, significant proportions reported having shouted at (90.6%), struck (69.1%) and slapped (39.3%) their children/child during the latest year as a measure of discipline. Seven percent used only the explanation method as a disciplinary measure. The proportion of mothers using the explanatory method predominantly went up in conjunction with increasing age (p<0.001) and higher educational achievement (p<0.001) (Table 1). On the other hand, the proportion of mothers using violent methods decreased with increasing age as evident in shouting at children (p<0.001), striking children (p<0.001) and slapping children (p<0.001) or any form of physical violence (p<0.001). Urban dwelling was associated with increased use of violent methods as evident in striking children (p<0.001), slapping children (p<0.001) and use of any form physical violence (p<0.001). Mothers with high education were less likely to use violent measures such as shouting at children (p<0.001), striking children (p<0.001), slapping children (p<0.001) and any form of physical violence against children (p<0.001). They were also more likely to use explanation predominantly as a measure of child discipline (p<0.001). A higher proportion of non-working women compared with working peers used violent methods such as striking children (p<0.001), slapping children (p<0.001) and any form of physical violence against children (p<0.001). They were also less likely to use explanation predominantly as a measure of child discipline (p<0.001) (Table 1).

**Table:** Table 1: **Number of women in each category (n), Proportions within each category of behavior against children (% of n), Adjusted Odds Ratios for behavior against children (OR) and Confidence Intervals of OR (CI) by demographic characteristics.**

Variable	(n)	Explain only to children		Shouted at children		Struck children		Slapped children		Any form of physical violence against children€	
		%	OR ( CI)	%	OR ( CI)	%	OR ( CI)	%	OR ( CI)	%	OR ( CI)
Age			1.1 (1.08 -1.12)#		0.93(0.92–0.95)#		0.91 (0.90–0.95)#		0.94 (0.93–0.95)#		0.91 (0.90–0.92)#
15-19 years	(28)	0		89		86		43		86	
20-24years	(1007)	2		95		83		52		84	
25-29years	(2725)	3		94		82		49		82	
30-34years	(2851)	4		94		79		46		79	
35-39years	(2972)	6		92		70		38		70	
40-44years	(2485)	10		87		56		30		56	
45-49years	(1948)	15		80		44		23		44	
Dwelling											
Urban	(5842)	7	0.61 (0.46–0.82)#	90	0.63(1.28–2.08)#	65	1.06 (0.91–1.24)	34	1.02 (0.88–1.18)	65	1.07 (0.92–1.26)
Rural	(8173)	6	1.0	91	1.0	72	1.0	43	1.0	72	
Education											
No education	(5300)	6	0.65 (0.39 - 1.05)	90	1.35 (0.89–2.06)	72	1.87 (1.42–2.47)#	45	2.37(1.77–3.19)#	72	1.87 (1.41–2.46)#
Primary	(2395)	8	0.72 (0.43 – 1.21)	90	1.31(0.83–2.05)	67	1.58 (1.17–2.12)**	40	2.13 (1.55–2.92)#	68	1.58 (1.18–2.13)**
Secondary	(5178)	6	0.96 (0.62 – 1.47)	92	1.24(0.84–1.81)	71	1.51 (1.18–1.93) #	37	1.53(1.16–2.02)**	71	1.53 (1.19–.96)#
Higher	(1142)	10	1.0	87	1.0	52	1.0	20	1.0	52	1.0
Current working status											
Not-working	(10652)	6	1.15 (0.85–1.58)	91	0.84 (0.64–1.10)	71	1.01 1.02(0.85–1.19)	41	0.99 (0.84–1.17)	72	1.01 1.02 (0.86–1.21)
Working	( 3334)	8	1.0	90	1.0	62	1.0	32	1.0	63	1.0

The adjusted odds ratio (OR) shown for each variable has been adjusted for all other variables (i.e. demographics, IPV indicators and attitudes towards wife beating). Age was used as a continuous variable in the regression. All other variables were used as categorical variables and the contrast category denoted with OR =1. 0. # p<0.001, **p<0.01, *p<0.05. € Either strike or slap or both.

**b. Demographic factors as predictors of maternal practices to correct child behavior:**

As indicated by the adjusted odds ratios (OR) (Table 1), increasing age was associated with a higher likelihood of predominantly using the explanation method and lower likelihood of using a violent method to correct child behavior. In addition urban dwellers were less likely to use the explanation method but more likely to use the violent method of shouting at children than their rural peers. Finally, women with secondary, primary and no education were more likely to use violent methods when contrasted with peers with a higher education as indicated in the adjusted odds ratios (Table 1).

**c. Proportion of mothers using specific practices to correct child behavior by exposure to and attitudes towards intimate partner violence:**

As indicated in Table 2, a lower proportion of women exposed to physical (p<0.001) and emotional (p<0.01) abuse when contrasted with non-abused peers, use the explanation method to correct child behavior. In addition, a lower proportion of women with tolerant attitudes towards wife abuse used the explanation method when contrasted with peers with non-tolerant attitudes (p<0.001). Women with experience of sexual violence more often used violent methods manifested in striking children (p<0.001), slapping children (p<0.001) and any form of physical violence against children (p<0.001). They were also less likely to use explanation predominantly as a measure of child discipline (p<0.001). The same trend was observed among women with experience of physical violence regarding shouting at children (p<0.001), striking children (p<0.001), slapping children (p<0.001) and any form of physical violence against children (p<0.001). Likewise, women with experience of emotional violence more often used violent methods manifested in shouting at children (p<0.05), striking children (p<0.001), slapping children (p<0.001) and any form of physical violence against children (p<0.001). They were also less likely to use explanation predominantly as a measure of child discipline (p<0.05).

**Table:** Table 2. **Number of women in each category (n), Proportions within each category of behavior against children (% of n), Adjusted Odds Ratios for behavior against children (OR) and Confidence Intervals of OR (CI) by exposure to violence and attitude to IPV.**

Variable	(n)	Explain only to children		Shouted at children		Struck children		Slapped children		Any form of physical violence against children€	
		%	OR ( CI)	%	OR ( CI)	%	OR ( CI)	%	OR ( CI)	%	OR ( CI)
Have sexual violence latest year											
No	(3908)	7	1.46 (0.84 – 2.54)	90	0.58 (0.37 -0.91)*	66	0.68 (0.52–0.88)**	36	0.64 (0.51–0.79)#	67	0.69 (0.53 – 0.90)**
Yes	(147)	3	1.0	95	1.0	81	1.0	55	1.0	81	1.0
Have physical violence latest year											
No	(3473)	7	0.61 (0.46 – 0.82)#	90	0.63 (1.28 – 2.08) #	65	1.06 (0.91 – 1.24)	34	1.02 (0.88 – 1.18)	65	1.07 (0.92 – 1.26)
Yes	(687)	6	1.0	91	1.0	72	1.0	43	1.0	72	
Have emotional violence latest year											
No	(3729)	7	1.33 (0.68-2.59)	90	0.98 (0.59–1.62)	68	0.88 (0.65–1.20)	37	0.80 (0.62 -1.03)	68	0.80 ( 0.58 – 1.10)
Yes	(431)	3	1.0	94	1.0	79	1.0	54	1.0	81	1.0
Attitude to wife beating											
Not justified	(6485)	8	1.34 (1.01 – 1.76)*	89	0.79 (0.62 -0.99)*	64	0.71 (0.61 – 0.83) #	32	0.71 (0.61-0.82)#	64	0.70 (0.60 – 0.81)#
justified	(7517)	5	1.0	92	1.0	74	1.0	45	1.0	74	1.0

The adjusted odds ratio (OR) shown for each variable has been adjusted for all other variables (i.e. demographics, IPV indicators and attitudes towards wife beating). All other variables were used as categorical variables and the contrast category denoted with OR =1. 0. # p<0.001, **p<0.01, *p<0.05. € Either strike or slap or both.

Finally, women with tolerant attitudes towards wife beating more often used violent methods manifested in shouting at children (p<0.001), striking children (p<0.001), slapping children (p<0.001) and any form of physical violence against children (p<0.001). They were also less likely to use explanation predominantly as a measure of child discipline (p<0.001).

**d. Experience of violence and attitudes towards wife abuse as predictors of practices to correct child behavior:**

As indicated by the adjusted odds ratios (OR) in Table 2, women’s non-tolerant attitude towards wife beating was associated with an increased likelihood of using the explanation method. Moreover, women’s experience of physical abuse was independently associated with an increased risk of shouting at children, striking children, slapping children and any use of physical violence against children. In addition non-tolerance of wife abuse among women was associated with a reduced risk of shouting at children, striking children, slapping children and any use of physical violence against children. Non-tolerance of wife beating also increased the likelihood of using the explanation method to correct child behavior.

## Discussion

This study investigated the association between intimate partner violence and practices used by mothers to discipline their children in the Egyptian context. Significant proportions of mothers used violent methods, like shouting, striking and slapping to correct child behavior. Seven percent of mothers used only the explanation option. Exposure to physical IPV and tolerant attitudes towards IPV were associated with an augmented risk of using violent methods (shouting, striking or slapping) to correct child behavior. Exposure to sexual and emotional violence however did not impact significantly on maternal practices to correct child behavior. Finally, non-tolerant attitudes towards IPV were associated with an increased likelihood of using only the explanation method.

An earlier study with a very small sample from Egypt tried to determine the prevalence of abusive behaviors among parents. The study tried to correlate child abuse with child and family characteristics.^48^ Other studies have previously indicated that intimate partner violence (IPV) is a risk factor for child physical abuse ^43,47^ though a distinction has not been made between the perpetrator and the victim of IPV as the child abuser. The current work adds to the literature by making this distinction, having the victim of IPV in focus. The findings indicate that the use of violent means to correct child behavior was common, with significant variation however depending on whether the mothers had themselves recently experienced an episode of spousal violence. Women’s experiences of physical violence and/or tolerant attitudes towards it increased the risk of them employing a violent approach to child discipline (i.e. shouting, striking or slapping) and reduced the likelihood of them using the explanation method in child discipline, at least in the Egyptian context. Though previous research did not study women exclusively or did not investigate factors associated with using non-violent methods in child discipline (i.e. explanation), these findings contribute to the growing literature indicating a cycle of violence from spousal to child abuse.^31,32,36-41,49^World studies of abuse in the family environment indicate that spousal violence against women is a common phenomenon in low-income countries including Egypt.^50,51^ So using the Egyptian context with nationally representative data, the current study indicates a strong relationship between maternal child abuse and mothers’ victimization of physical violence.

The current work provides some new insight in the research concerning the hypothesized association between spousal emotional and sexual abuse and parental violent abuse of their children. Women with an experience of spousal emotional and sexual abuse used violent methods to correct child behavior more often than non-abused peers. However, these results could not be statistically verified when possible confounding variables were adjusted for in the logistic regression model. These findings are rather difficult to reconcile considering that physical IPV remained a significant factor in explaining child abuse even when confounding was corrected for in a logistic regression. A plausible explanation could be that if women consider spousal abuse acceptable, they may be more prone to accept abuse of their children. Data emerging from different parts of Africa, including Egypt, indicate that significant proportions of women consider physical wife abuse an acceptable social norm.^52,53^ There is no evidence however that sexual and emotional wife abuse is an acceptable societal norm in the African contexts. Perhaps, this could explain why physically abused women are more prone to abuse their children, an observation not apparent among emotionally or sexually abused women. Further research linking different facets of spousal abuse to child abuse may need to scrutinize societal norms surrounding the acceptability of the different types of spousal violence in that specific society.

The current work incorporated the contribution of possible confounder variables (i.e. age, education, rural/urban dwelling and working status) in explaining methods used to correct child behavior. With a few exceptions, the results were mainly contradictory to previous work. Our findings suggested augmented risk for child abuse as parental age increased thus corroborating some studies^47^ but contradicting others where no association was observed.^54^ Further, our study suggests that a higher educational level among mothers may reduce the risk of child abuse contradicting the findings of previous work where higher parental education was associated with an increased likelihood of child abuse.^34,55^ The discrepancies in findings may result from differences in sample characteristics. The current work investigated women exclusively, while others have either observed only men exclusively^34,37^ or have not differentiated between men and women.^43^ Moreover, issues of sample size may also have limited the possibility of previous work to detect statistically significant differences. Another plausible explanation for the discrepancy in findings regarding determinants could stem from differences in the societal context in which the studies are performed. Beliefs, religion and attitudes towards child upbringing among other things vary between different societies. In some societies, for example, shouting at, slapping or striking children is a societal norm, indicating that such practices may occur frequently, irrespective of parental age, education or social status. Scrutiny of the mechanism linking contextual factors to child abuse in each unique societal context is therefore crucial for research in the field.

This difficulty of assigning causality when a cross-sectional design is used warrants acknowledgement. For example, it is unclear whether spousal abuse predisposed child abuse or whether it was a consequence of child abuse. Secondly, though attempts where made to account for confounding, residual confounding of contextual variables (e.g. poverty) could not be controlled. Though Egypt is in a transitional phase of socioeconomic development, the society may still be fragmented with regard to the socioeconomic status of women. One can also question the response rate of over 99 percent. As regards questionnaires to the general public, response rates tend to be much lower. However, the current DHS survey (EDHS, 2005) is the ninth in the series, suggesting that interviewers might have gained sufficient experience to conduct interviews of this nature. This is likely to improve the response rate over time.

Despite these limitations, we, thus recommend the implementation of local parental education programs focusing on communicative skills to reduce IPV and related child abuse. In addition, interventions aimed at changing women’s attitudes towards wife abuse and reducing exposure to IPV are likely to modify maternal risk behavior of abusing their child while promoting a non-violent approach to correct child behavior. Future researchers should acknowledge the contribution of the societal context when studying risk factors for child abuse.

## References

[1] Springer K, Sheridan J, Carnes D (2003). The long-term health outcomes of childhood abuse: An overview and a call to action.. Journal of General Internal Medicine..

[2] Nelson EC, Heath AC, Madden PA (2002). Association between self-reported childhood sexual abuse and adverse psychosocial outcomes: results from a twin study.. Archives of General Psychiatry..

[3] Kessler RC, Davis CG, Kendler KS (1997). Childhood adversity and adult psychiatric disorder in the U.S. National Comorbidity Survey.. Psychological Medicine..

[4] Jumper SA (1995). A meta-analysis of the relationship of child sexual abuse and psychological adjustment.. Child Abuse and Neglect.

[5] World Health Organization. World report on violence and health. Geneva: WHO, 2002.

[6] UNO. 2006 http://www.violencestudy.org/IMG/pdf/ English.pdf, accessed May 27 2007.

[7] Youssef RM, Attia MS, Kamel MI (1998). Children experiencing violence: parental use of corporal punishment.. Child Abuse and Neglect..

[8] Hahm H, Guterman N (2001). The emerging problem of physical child abuse in South Korea.. Child Maltreatment..

[9] Browne K (2002). Child abuse and neglect in Romanian families: a national prevalence study 2000.. Copenhagen: WHO Regional Office for Europe.

[10] Ketsela T, Kedebe D (1997). Physical punishment of elementary school children in urban and rural communities in Ethiopia.. Ethiopian Medical Journal..

[11] McCauley J, Kern DE, Kolodner K (1997). Clinical characteristics of women with a history of childhood abuse: unhealed wounds.. JAMA.

[12] Springs FE, Friedrich WN (1992). Health risk behaviours and medical sequelae of childhood sexual abuse.. Mayo Clin Proceedings..

[13] Walker EA, Keegan D, Gardner G, Sullivan M, Bernstein D, Katon WJ (1997). Psychosocial factors in fibromyalgia compared with rheumatoid arthritis: II. Sexual, physical, and emotional abuse and neglect.. Psychosomatic Med..

[14] Weiss E, Longhurst J, Mazure C (1999). Childhood sexual abuse as a risk factor for depression in women: psychological and neurobiological correlates.. Am J Psychiatry..

[15] Kessler RC, Magee WJ (1994). Childhood family violence and adult recurrent depression.. J Health Soc Behav..

[16] Saunders BE, Villeponteaux LA, Lipovsky JA, Kilpatrick DG, Veronen LJ (1992). Child sexual assault as a risk factor for mental disorders among women.. J Interpers Violence..

[17] Kendler KS, Bulik CM, Silberg J, Hettema JM, Myers J, Prescott CA (2000). Childhood sexual abuse and adult psychiatric and substance use disorders in women.. Arch Gen Psychiatry..

[18] Heim C, Ehlert U, Hanker JP, Hellhammer DH (1998). Abuse-related posttraumatic stress disorder and alterations of the hypothalamic-pituitary-adrenal axis in women with chronic pelvic pain.. Psychosomatic Med..

[19] Heim C, Owens MJ, Plotsky PM, Nemeroff CB (1997). The role of early adverse life events in the etiology of depression and posttraumatic stress disorder. Focus on corticotropin-releasing factor.. Ann N Y Acad Sci..

[20] Walling MK, O'Hara MW, Reiter RC, Milburn AK, Lilly G, Vincent SD (1994). Abuse history and chronic pain in women: II. A multivariate analysis of abuse and psychological morbidity.. Obstet Gynecol..

[21] Jamieson DJ, Steege JF (1997). The association of sexual abuse with pelvic pain complaints in a primary care population.. Am J Obstet Gynecol..

[22] Laws M (1993). Does a history of sexual abuse in childhood play a role in women's medical problems: a review.. J Women’s Health..

[23] Van Houdenhove B, Neerinckx E, Lysens R, Vertommen H, Van Houdenhove L, Onghena P, Westhovens R, D'Hooghe MB (2001). Victimization in chronic fatigue syndrome and fibromyalgia in tertiary care: a controlled study on prevalence and characteristics.. Psychosomatics..

[24] Boisset-Pioro MH, Esdaile JM, Fitzcharles MA (1995). Sexual and physical abuse in women with fibromyalgia syndrome.. Arthritis Rheum..

[25] Drossman DA, Leserman J, Nachman G (1990). Sexual and physical abuse in women with functional or organic gastrointestinal disorders.. Ann Intern Med.

[26] Leserman J, Zhiming L, Drossman DA, Toomey TC, Nachman G, Glogau L (1997). Impact of sexual and physical abuse dimensions on health status: development of an abuse severity measure.. Psychosomatic Med..

[27] Felitti VJ, Anda RF, Nordenberg D, Williamson DF, Spitz AM, Edwards V, Koss MP, Marks JS (1998). Relationship of childhood abuse and household dysfunction to many of the leading causes of death in adults: the Adverse Childhood Experiences (ACE) Study.. Am J Prev Med..

[28] Thompson M, Arias I, Basile K, Desai S (2002). The association between childhood physical and sexual victimization and health problems in adulthood in a nationally representative sample of women.. J Interpers Violence..

[29] Finestone HM, Stenn P, Davies F, Stalker C, Fry R, Koumanis J (2000). Chronic pain and health care utilization in women with a history of childhood sexual abuse.. Child Abuse and Neglect..

[30] Anda RF, Croft JB, Felitti VJ (1999). Adverse childhood experiences and smoking during adolescence and adulthood.. JAMA.

[31] Bethea L (1999). Primary prevention of child abuse.. American Family Physician..

[32] Straus M, Gelles R, Steinmetz S (1990). Physical violence in the American family.. Beverly Hills, CA: Sage Publications..

[33] Cicchetti D, Rizley R (1981). Developmental perspectives on the etiology, integrational transmission and sequalae of child maltreatment.. New Directions for Child Development..

[34] Bowker L, Arvitell M, McFerrom J (1988). On the relationship between wife beating and child abuse. In K Yllo and M Bograd (Eds.), Feminist Perspectives on Wife Abuse. Newbury Park, CA: Sage.

[35] Straus M, Smith C (1990). Family patterns and child abuse. In M. A. Straus & R. J. Gelles (Eds.), Physical violence in American families: Risk factors and adaptations to violence in 8,145 families.. New Brunswick, NJ: Transaction Publishers..

[36] Gayford J (1975). Wife battering: a preliminary survey of 100 cases.. BMJ.

[37] Giles-Sims S (1985). A longitudinal study of battered children of battered wives.. Family Relations..

[38] McKibben L, De Vos E, Newberger E (1989). Victimization of mothers of abused children: a controlled study.. Pediatrics..

[39] Ross S (1996). Risk of physical abuse to children of spouse abusing parents.. Child Abuse and Neglect..

[40] Stark E, Flitcraft A (1998). Women and children at risk: a feminist perspective on child abuse.. Int J Health Serv..

[41] Straus M, Gelles R, Steinmetz S (1980). Behind closed doors.. Garden City, NY: Anchor Press..

[42] McKay MM (1994). The link between domestic violence and child abuse: assessment and treatment considerations.. Child Welfare..

[43] Tazima E (2000). The relative importance of wife abuse as a risk factor for violence against children.. Child Abuse and Neglect.

[44] Dubowitz H, Black M, Kerr M (2001). Type and timing of mothers' victimization: effects on mothers and children.. Pediatrics..

[45] Strauss MA (1990). Measuring intra-family conflict and violence: The conflict tactics (CT) scales. In MA Strauss & RJ Gelles (Eds.): Physical violence in American families: Risk factors and adaptations to violence in 8145 families.. New Brunswick, NJ: Transaction.

[46] World Health Organization. Putting Women First: Ethical and Safety Recommendations for Research on domestic Violence against Women. Geneva: WHO, 2005.

[47] Straus MA (1994). Beating the devil out of them: Corporal punishment by parents and its effects on children.. New York: Lexington Books..

[48] Hassan F, Refaat A, El-Sayed H, El-Defrawi H (1999). Disciplinary practices and child maltreatment among Egyptian families in an urban area in Ismailia city.. The Egyptian Journal of Psychiatry..

[49] Dalal K, Rahman F, Jansson B (2008). The origin of violent behaviour among child labourers in India.. Global Public Health..

[50] Hassan V, Sadowski L, Bangadiwala S, Vizcarra B, Ramiro L, De Paula C, Bordin I, Mitra M (2004). Physical Intimate Partner Violence in Chile, Egypt, India and the Phillippines.. Injury Control and Safety Promotion..

[51] Jeyaseelan L, Sadowski L, Kumar S, Hassan F, Ramiro L, Vizcarra B (2004). World studies of abuse in the family environment – risk factors for physical intimate partner violence.. Injury Control and Safety Promotion..

[52] Lawoko S (2006). Factors associated with attitudes towards intimate partner violence: A study of women in Zambia.. Violence and Victims..

[53] Egypt DHS. 2005: http://www.measuredhs.com/pubs /pub_details.cfm?ID=586&ctry_id=10&SrchTp=ctry#dfils, accessed May 27 2007.

[54] Cappelleri JC, Eckenrode J, Powers JL (1993). The epidemiology of child abuse. Findings from the Second National Incidence and Prevalence Study of Child Abuse and Neglect.. Am J Public Health..

[55] Margolin L, Larson O (1988). Assessing mothers’ and fathers’ violence towards children as a function of their involuntary participation in family work.. Journal of Family Violence..

